# Antioxidant Effects of Cactus Seed Oil against Iron-Induced Oxidative Stress in Mouse Liver, Brain and Kidney

**DOI:** 10.3390/molecules29184463

**Published:** 2024-09-20

**Authors:** Habiba Bouchab, Abbas Ishaq, Youness Limami, Gabriele Saretzki, Boubker Nasser, Riad El Kebbaj

**Affiliations:** 1 Laboratory of Health Sciences and Technologies, Higher Institute of Health Sciences, Hassan First University of Settat, Settat 26000, Morocco; habibabouchab78@gmail.com (H.B.); youness.limami@gmail.com (Y.L.); 2 Higher Institute of Nursing Professions and Technical Health (ISPITS), Errachidia 52000, Morocco; 3 Laboratory of Biochemistry, Neurosciences, Natural Resources and Environment, Faculty of Science and Technology, Hassan First University of Settat, Settat 26000, Morocco; boubker.nasser@uhp.ac.ma; 4 Biosciences Institute, Newcastle University, Campus for Ageing and Vitality, Newcastle upon Tyne NE2 4HH, UK; abbas.ishaq@gmail.com (A.I.); gabriele.saretzki@ncl.ac.uk (G.S.)

**Keywords:** oxidative stress, antioxidant enzymes, ferrous sulfate, liver, brain, kidney, DNA damage

## Abstract

In recent times, exploring the protective potential of medicinal plants has attracted increasing attention. To fight reactive oxygen species (ROS), which are key players in hepatic, cerebral and renal diseases, scientists have directed their efforts towards identifying novel compounds with antioxidant effects. Due to its unique composition, significant attention has been given to Cactus Seed Oil (CSO). Iron, as a metal, can be a potent generator of reactive oxygen species, especially hydroxyl radicals, via the Fenton and Haber–Weiss reactions. Here, we employed ferrous sulfate (FeSO4) to induce oxidative stress and DNA damage in mice. Then, we used CSO and Colza oil (CO) and evaluated the levels of the antioxidants (superoxide dismutase [SOD], glutathione peroxidase [GPx] and glutathione [GSH]) as well as a metabolite marker for lipid peroxidation (malondialdehyde [MDA]) relating to the antioxidant balance in the liver, brain and kidney. In addition, we measured DNA damage levels in hepatic tissue and the effects of CSO on it. Our study found that iron-dependent GPx activity decreases in the liver and the kidney tissues. Additionally, while iron decreased SOD activity in the liver, it increased it in the kidney. Interestingly, iron treatment resulted in a significant increase in hepatic MDA levels. In contrast, in brain tissue, there was a significant decrease under iron treatment. In addition, we found varying protective effects of CSO in alleviating oxidative stress in the different tissues with ameliorating DNA damage after iron overload in a mouse liver model, adding compelling evidence to the protective potential of CSO.

## 1. Introduction

Reactive oxygen species refer to unstable and reduced forms of oxygen derivatives. Some examples of ROS include hydrogen peroxide (H_2_O_2_), superoxide anion (O_2_^−•^) and hydroxyl radical (OH^•^) [1]. The ROS is physiologically generated during mitochondrial respiration and by other enzymes such as NADH oxidases (NOXes). The ROS controls the proliferation of cells, pro-fibrotic signalling, cell death and pro-inflammatory signalling [2]. In contrast, at a substantial level of production overwhelming the antioxidant defence capacities, they result in oxidative stress. Antioxidants are divided into enzymatic such as superoxide dismutase (SOD), glutathione peroxidase (GPx), and catalase (CAT), and non-enzymatic such as glutathione (GSH), ascorbic acid, thioredoxines, and many more [3]. Oxidative stress can be triggered by various metals, including iron, with the consequence of ROS generation [4]. Iron is a vital component of human metabolism and health. However, an excess of it causes iron overload, thereby contributing to the development of several diseases, such as chronic liver damage [5] because most iron is stored in the liver in a ferritin-bound fashion [6]. Iron generates reactive oxygen species, especially hydroxyl radicals, via the Fenton and Haber–Weiss reactions [7], which trigger oxidative damage to lipids, proteins, and DNA [2]. A recognized relationship exists between oxidative stress and DNA damage [8]. In fact, it has been reported that hydroxyl radicals attach to the carbon of positions 4, 5, and 8 of purines in the DNA, producing OH- adduct radicals. In addition, two known OH- adducts are formed for adenine, especially at the 4th and 8th positions [9]. Three known iron chelators have been synthesized to treat various diseases associated with iron overload, including deferoxamine, deferiprone and deferasirox [10]. However, these drugs showed unwanted or even dangerous side effects [11]. This issue prompts scientists to explore new compounds with antioxidant potential derived from traditional plant-based medicine. The potential of antioxidants in counteracting the deleterious effects of reactive oxygen species have been well-established [12]. In this context, we and other research teams have primarily focused on exploring the biological potential of plant-derived oils. We hypothesis that some plants with beneficial properties for health, such as cactus seed oil, may be based on protective effects on oxidative stress and associated factors. Cactus seed oil (CSO) can be obtained from the prickly pear cactus (*Opuntia ficus-indica*) [13]. The Nopal cactus has been used for centuries in nutrition, health, and cosmetics as jam, tea, juice, and extracted oil. Moreover, cactus flowers and fruits are routinely used in the Sub-Saharan area as anti-diarrheal and anti-ulcerogenic agents [12]. Chemical analyses reveal that CSO predominantly consists of linoleic acid (57.72–63.11%) and oleic acid (19.37–21.79%) [14]. In contrast, CO (Colza Oil) primarily contains oleic acid (63%) and an intermediate proportion of linoleic acid (19%) [15]. In addition, other fatty acids were identified in intermediate or minimal quantities in both oils such as palmitic acid (12.47–15.06% in CSO versus 4.5% in CO), stearic acid (2.56–4.12% in CSO versus 1.6% in CO), arachidic acid (0.19–0.21% in CSO versus 0.5% in CO) and eicosenoic acid (0.09–0.12% in CSO versus 1.3% in CO) [14,15]. In addition, analyses showed the richness of CSO in γ-tocopherol, ranging from about 315.47 mg/kg to 502.04 mg/kg [16]. In contrast, CO contains approximately 69.01 mg/kg [15]. Moreover, CSO and CO have a common composition of β-sitosterol [15,17]. Importantly, CSO contains two major phytosterols (schottenol and spinasterol) [18], which are entirely absent in CO [15]. This reason is why CSO is considered distinctive. To our knowledge, there have been no prior studies about the effects of CSO and CO from plants grown in Morocco on iron overload in mice, highlighting the original nature of our investigation. Therefore, we investigated the antioxidant effects of CSO and CO on iron-induced oxidative stress in mice. The following three organs were chosen in this present work: liver, brain, and kidney. These organs were selected because of their sensitivity to iron overload and oxidative stress [19,20,21]. Our study focused on parameters of oxidative stress and DNA damage (SOD, GPx, MDA, GSH, and γH2A.X). The potential protective effects of CSO against iron were compared to CO. The CO is another common food frequently consumed in Europe [22]. We found rather variable effects of all treatments (iron and oils) in the 3 analysed tissues.

## 2. Results

### 2.1. Opuntia ficus-indica Seed Oil Composition

Table 1 summarizes the data of the chemical analysis of CSO and CO. The four major fatty acids were linoleic acid (ω6; C18:2), palmitic acid (C16:0), oleic acid (C18:1) and stearic acid (C18:0), representing 67.32%, 13.85%, 9.95% and 4.61%, respectively. The other fatty acids are present in minimal quantities. The CO primarily contains oleic acid (C18:1), palmitic acid (C16:0), eicosenoic acid (C20:1) and arachidic acid (C20:0) representing 77.18%, 6.48%, 4.84% and 2.38%, respectively. In addition, the Table revealed that *Opuntia ficus-indica* seed oil contains γ-tocopherol (0.08% versus 0.15% in CO) as major tocopherol. The studied oil also contains γ-sitosterol as a major phytosterol, representing 0.92%. On the other hand, CO contains γ-sitosterol and campesterol representing 1.77% and 1.35%, respectively.

### 2.2. Effects of CSO and CO on MDA Content in the Liver, Brain and Kidney of Iron-Overloaded Mice

**Figure 1:**
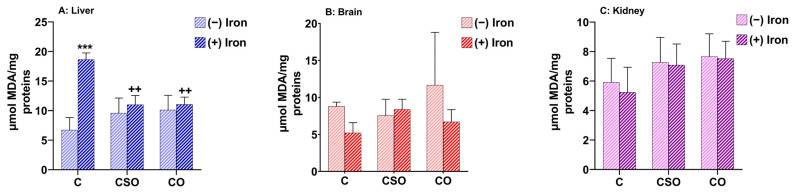
MDA levels in mouse liver, brain and kidney upon treatment with iron, CSO and CO. All values are means ± SD (n = 3–4 mice/group). *** *p* ˂ 0.001 compared to untreated control. ^++^ *p* ˂ 0.01 compared to iron-treated samples. Statistics were performed using two-way ANOVA followed by Tukey’s test for multiple comparisons.

Lipids play a prominent role in cell homeostasis since they act as signalling molecules, serve as structural components of cell membranes and function as energy stores [23]. Polyunsaturated fatty acid residues of lipids are sensitive to oxidation, and this sensitivity increases as a function of the number of double bonds per fatty acid molecule [24]. Lipid peroxidation is a common outcome of oxidative stress, where ROS initiate the oxidation of lipids [25]. The MDA is the best-known end-product generated during lipid peroxidation [26]. The antioxidant capacity of CSO and CO treatments on MDA levels was studied in the liver, brain and kidney of experimental animals upon iron treatment. The oxidative stress marker, MDA, was quantified in these three tissues from the different experimental mouse groups under control and iron-related conditions. Interestingly, iron treatment resulted in a significant increase in hepatic MDA levels compared to untreated mice (*p* ˂ 0.001) (Figure 1A). In contrast, in both brain and kidney tissues, there was no change under any treatment condition (Figure 1B,C). Importantly, treatment with CSO (*p* ˂ 0.01) or CO (*p* ˂ 0.01) maintained hepatic MDA content near the control level in iron-treated samples (Figure 1A). Thus, the expected effects were found in the liver, but no effects of iron or oils occurred in the other two tissues.

### 2.3. Effects of CSO and CO on GSH Content in the Liver, Brain and Kidney of Iron-Overloaded Mice

**Figure 2:**
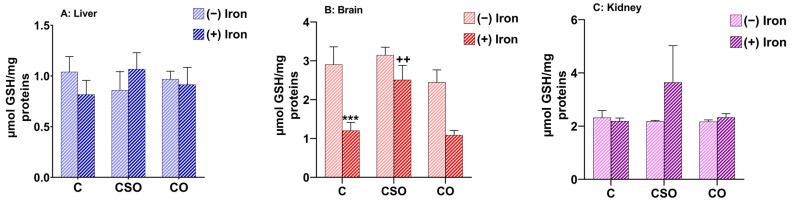
GSH level related to CSO and CO treatments in mouse liver, brain and kidney in controls and upon iron treatment. All values are means ± SD (n = 3–4 mice/group). *** *p* ˂ 0.001 compared to untreated control. ^++^ *p* ˂ 0.01 compared to iron-treated samples. Statistics were performed using two-way ANOVA followed by Tukey’s test for multiple comparisons.

The GSH is an antioxidant that plays a key role in maintaining physiological functions and homeostasis of redox balance. The antioxidant capacity of CSO and CO treatments on GSH levels was studied in the liver, brain and kidney of experimental animals in controls and upon iron treatment. While iron treatment itself did not show any variation in hepatic and renal glutathione levels (Figure 2A,C), it decreased the cerebral GSH amount by more than half (*p* ˂ 0.001) (Figure 2B). Either oils alone or combined with iron were not able to affect the GSH level in the kidney and liver (Figure 2A,C). In contrast, CSO was able to normalize the cerebral GSH content to the level observed in the control mice (*p* ˂ 0.01) while CO had no protective effect compared to untreated controls (Figure 2B). In summary, effects of iron treatment on GSH were only found in the brain where CSO was able to counteract the adverse effect of iron.

### 2.4. Effects of CSO and CO on SOD Activity in the Liver, Brain and Kidney of Iron-Overloaded Mice

**Figure 3:**
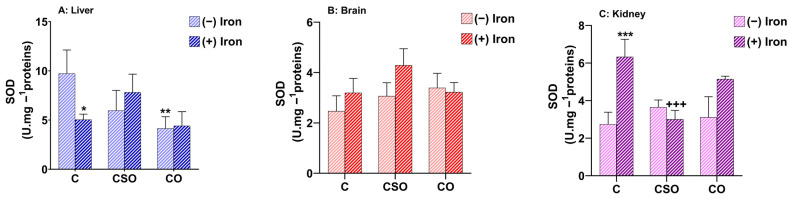
SOD activity of CSO and CO in mouse liver, brain and kidney upon iron treatment. All values are means ± SD (n = 3–4 mice/group). *** *p* < 0.001, ** *p* < 0.01, * *p* < 0.05 compared to untreated control. (^+++^ *p* ˂ 0.001) compared to iron-treated samples. Statistics were performed using two-way ANOVA followed by Tukey’s test for multiple comparisons.

Superoxide dismutases (SODs) are the first line of defence against reactive oxygen species which can be induced by iron treatment which is known to exert lipid peroxidation [27]. There are three forms of SOD with two of them (SOD 1 and SOD3) using Cu/Zn and one, MnSOD (SOD2), using manganese to catalyse the dismutation of a superoxide anion to molecular oxygen and hydrogen peroxide. These enzymes protect cells against the cytotoxic effects of the highly reactive oxygen free radical superoxide [28]. The antioxidant capacity of CSO and CO on SOD levels was studied in the liver, brain and kidney of control and iron-treated animals. Iron treatment significantly decreased the activity of SOD in the liver (*p* < 0.05) and, intriguingly, doubled SOD activity in the kidney (*p* < 0.001). We did not detect any effect of iron-stress in brain tissue (Figure 3A–C). An effect of decreased SOD levels for CO supplementation in iron-untreated samples was found in the liver compared to the oil-untreated control. In addition, there was no effect for both oils on SOD activity under the condition of iron treatment in this tissue type (Figure 3A). In both the brain and kidney, there was no significant effect on superoxide dismutase activity between the iron treatment and control groups under CO treatment. Similarly, there was no effect of CSO under any treatment condition in the liver (Figure 3A). Interestingly, in the kidney, an iron-dependent increase in SOD activity in the control tissue was abolished by treatment with cactus seed oil (*p* < 0.001) (Figure 3C). No significant changes were found in cerebral SOD under any condition (Figure 3B). Thus, iron- and oil-dependent effects were rather heterogenous in the three tissues and did not show any consistent or protective effects.

### 2.5. Effects of CSO and CO on GPx Activity in the Liver, Brain and Kidney of Iron-Overloaded Mice

**Figure 4:**
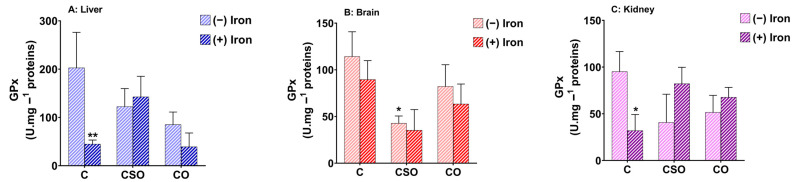
Measurement of GPx activity of CSO and CO treatments in mouse liver, brain and kidney upon iron treatment. All values are means ± SD (n = 3–4 mice/group). ** *p* < 0.01, * *p* < 0.05 compared to untreated control. Statistics were performed using a two-way ANOVA followed by Tukey’s test for multiple comparisons.

The determination of the cell fate depends on maintaining the cellular redox function. Glutathione peroxidases (GPxs) are a prominent family of eight antioxidant enzymes for the maintenance of redox balance by combating oxidative stress. The GPx catalyses the reduction of hydrogen peroxide and/or hydroperoxides to water or corresponding alcohols [29]. The GPx enzymes use glutathione in the reduction of H_2_O_2_. The catalytic antioxidant activity of GPx was measured in three organs from different experimental mouse groups. In the liver and kidney, GPx activity was strongly decreased by iron administration compared to the control group (Figure 4A,C). While there was no effect of iron in brain tissue, a decrease in GPx activity was found due to CSO supplementation with and without iron treatment (Figure 4B). In contrast, no significant changes were observed in any of the remaining treatment groups although there were some trends towards a counteraction of decreased GPx activity after iron treatment in the liver and kidney (Figure 4A–C). Thus, while iron decreased GPX activity in two of the three tissues, the oils only showed trends to counteract this treatment without reaching statistical significance.

### 2.6. Effects of CSO and CO on DNA Damage in the Liver of Iron-Overloaded Mice

**Figure 5:**
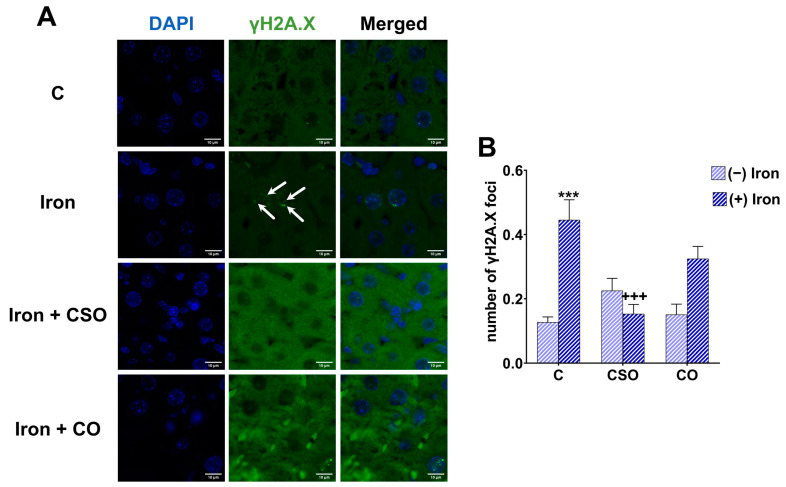
Effects of CSO and CO treatments on DNA of mouse liver. (**A**) Representative images of γH2A.X foci in liver tissue. Blue: DAPI, green: γH2A.X foci. Images were acquired at 400× magnification. γH2A.X foci are marked by arrows. (**B**) Average γH2A.X focus number per nucleus. Five images were taken at different fields of view for each sample. All values are means ± SEM (n = 3 mice/group). *** *p* ˂ 0.001 compared to untreated control. ^+++^ *p* ˂ 0.001 compared to iron-treated samples. Statistics were performed using two-way ANOVA followed by Tukey’s test for multiple comparisons.

Using γH2A.X as a biomarker for DNA damage on paraffin-embedded liver sections, we analysed the average number of DNA damage foci in this tissue (Figure 5A). The CSO and CO administration did not have any effect on their own on the average number of DNA damage foci compared to control sections. In contrast, iron significantly enhanced the DNA damage approximately three times compared to the control group (*p* < 0.05). Importantly, the combined treatment of CSO with iron showed a significant decrease in the number of γH2A.X foci, which demonstrated a clear protective effect compared to the iron-overload group (Figure 5B).

## 3. Discussion

Although there are numerous studies discussing the antioxidant potential of cactus seed oil using chemical tests, to date, few studies have explored its ability to prevent oxidative stress in animal models. The aim of the current study was to investigate the protective properties of CSO and CO by analysing several oxidative stress-related parameters including two antioxidant enzyme groups. The production of free radicals within physiological limits is a fundamental mechanism that contributes to signalling, proper functioning and survival of cells. However, when there is an imbalance between the production and the neutralization of ROS by antioxidants, oxidative stress is induced which causes damage to cell membranes, lipids, proteins, lipoproteins and DNA [2]. To counteract this oxidative stress, cells are equipped with antioxidants, categorized into enzymatic and non-enzymatic systems [3]. In cases of iron overload within tissues, iron induces oxidative stress through the Fenton reaction [30]. While the liver is the organ responsible for iron regulation and storage [31], iron overload can cause chronic liver damage which can even progress towards liver cancer [32]. Our results showed that iron induced lipid peroxidation (MDA) in liver tissue accompanied by decreased SOD and GPx activity levels. In contrast, iron supplementation had no effect on GSH level in this tissue. These results correspond well to data from other studies showing that iron led to a significant reduction of SOD and GPx activities and induction of MDA level [33]. It is well known that the hydroxyl radical is an initiator of lipid peroxidation [25] and a powerful oxidant generated via the Fenton reaction [34]. Importantly, we found that treatment with CSO and CO was able to prevent the effects of iron on MDA production in liver tissue. Both CSO and CO have β-sitosterol in common in their composition [15,17], something that might be responsible for the reduction of malondialdehyde overproduction during liver injury [35]. In addition, it was observed that dietary β-sitosterol increased the jejunal NRF2 mRNA level [36], which is consistent with other studies demonstrating that this phytosterol upregulated NRF2 mRNA levels and alleviated oxidative damage in vivo and in vitro [37,38,39]. Given the relationship between NRF2 and oxidative stress, it has been well-explained in a recent review how components such as tocopherols; sterols, especially schottenol and spinasterol; ferulic acid; and fatty acids could modify NRF2 mRNA levels following oxidative stress [40].

It was shown by others that iron overload in the brain is linked to the development of neurodegenerative diseases including Alzheimer’s disease, Parkinson’s disease, Friedreich’s ataxia and Huntington’s disease [41]. However, the exact mechanism of how iron overload causes neurodegenerative diseases remains to be fully understood [41]. Our results showed that neither MDA levels nor SOD and GPx activity levels in the brain of mice were affected by iron treatment. In contrast, we found that iron supplementation significantly decreased GSH level in the brain. Similarly, a decline in the amount of GSH is a common finding in patients with neurodegenerative diseases such as Parkinson’s disease and Alzheimer’s disease and can even cause neurodegeneration prior to disease onset [42,43,44]. Intriguingly, patients with Parkinson’s disease exhibit elevated iron levels [45]. This finding paves the way for novel avenues of investigation within the research field of neurodegenerative diseases. We discovered in this study that CSO supplementation was able to effectively counterbalance the effects of iron overload on GSH levels in the brain. Together with the above-described findings on the significance of GSH levels for several neurodegenerative diseases, our results of the beneficial effects of CSO on GSH levels in the brain after iron treatment might be encouraging for further studies on the possible therapeutic value of this oil. However, it should be noted that CSO increased GPx activity levels even without iron supplementation, so it is not entirely clear whether it truly counteracted iron stress or already has a general protective effect in this tissue.

Kidney tissue exhibited lower sensitivity to iron overload compared to liver tissue. In fact, we did not find any effects of iron treatment on MDA or GSH levels. Others also found no increase in MDA after dietary iron application in rats [46]. Surprisingly, we detected a substantial (around two-fold) increase in SOD activity within the renal tissue of mice subjected to iron overload while the above study found such an increase in the liver, but not in the kidney [46] demonstrating that such a counteraction to iron-induced oxidative stress by selected antioxidant enzymes is a possible scenario. In contrast, GPx activity was decreased similar to that observed in the liver. The increase in SOD activity might presumably be a tissue-specific reaction as a response to increased ROS generated from the iron-induced stress although it is not clear why it is different from other tissue types. In contrast, the decrease in GPx activity follows the expected effect in the kidney.

Based on our current results, it appears that iron as an oxidizing agent affects the three analysed organs at different levels. Likewise, CSO has exhibited some protective potential by moderating oxidative stress to different extents. All three organs play important roles to maintain overall health. However, their functions differ significantly. Liver performs a diverse range of functions, which include carbohydrate and lipid homeostasis, maintenance of metal homeostasis, removal of infectious agents via Kupffer cells and detoxification of blood [47]. The central role of the brain is to perform cognitive functions, regulate emotions and process information [48]. On the other hand, the primary role of kidneys is the filtration of blood and removal of waste products in the urine to maintain electrolyte balance and regulate blood pressure [49]. In addition, the brain relies on a continuous supply of oxygen for optimal functioning, requiring 20% of the body’s total oxygen consumption [50]. In contrast, the kidneys, which require oxygen for their vital processes, have a comparatively lower demand of approximately 7% [51]. In fact, oxygen metabolism in mitochondria is the main generator of ROS [52]. It has been noted that moderate hyperbaric conditions can enhance ATP levels in the kidney and liver; extreme oxygen exposure leads to critical reductions in ATP across the kidney, liver and brain, with the brain being the most vulnerable to oxygen toxicity [53]. The levels of various macroelements (Na, Mg, P, S, K, Ca) and microelements (Fe, Mn, Co, Cu, Zn, Se, I, As, Cd, Hg, Pb, Li, B, Sr) in the liver, kidney and brain of rats vary significantly [54]. In fact, iron as an example is a microelement able to form ROS through the Fenton and Haber–Weiss reaction [55]. In summary, the divergence in the results observed between the three organs might be due to their distinct functions and metabolic activities including that of oxygen.

The putative protective effect of both oils, but especially CSO, is most likely due to their antioxidative potential. In fact, analyses showed a high content of γ-tocopherol in CSO, ranging from about 315.47 mg/kg to 502.04 mg/kg [16], whereas CO contains only approximately 69.01 mg/kg [15]. Moreover, both CSO and CO have β-sitosterol in their composition [15,17]. Importantly, CSO contains two major phytosterols (schottenol and spinasterol) [18] which are entirely absent in CO [15]. These differences might be the reason for the differential effects of both oils. Additionally, the CSO contains a high quantity of linoleic acid (71.59%) [16] which may have antioxidative potential. Various studies have demonstrated significant antioxidative properties of conjugated linoleic acid (CLA) and linoleic acid derivative (DCP–LA). The CLA has shown antioxidative potential in rat brains, as evidenced by GSH level, GPx, MnSOD, complex I and complex IV activities [56]. In addition, CLA supplementation has also been noted to be beneficial in managing chronic obstructive pulmonary disease (COPD) by inhibiting oxidative stress [57]. In lactating dairy cows, CLA in the chosen formulation, application period and dosage had a marginal antioxidative potential in terms of lipid peroxidation [58]. The DCP–LA has been noted to protect against oxidative stress-induced apoptosis highlighting its capacity to reduce cell death in stress-related conditions [59]. Given that Nrf2 plays a key role as a transcription factor in the defence against oxidative stress, an association between the Nrf2 signalling pathway and antioxidants could be suggested. In fact, Roselle seed oil, rich in linolenic (30%) and oleic acids (14.45%), enhances Nrf2 levels in rat livers exposed to paracetamol [60]. These findings collectively underscore the potential effect of LA and its derivatives in mitigating oxidative stress.

Iron participates in the generation of ROS, in particular the hydroxyl radical due to the Fenton reaction that cause high levels of damage to cells and tissues [5,55]. The hydroxyl radical is the most reactive oxygen species toward DNA [61]. In this study, DNA damage was evaluated with an immunofluorescence technique using γH2A.X as a biomarker. Due to its sensitivity, γH2A.X is widely used in clinical and research studies [62]. We found that iron treatment enhanced DNA damage in liver tissue by increasing the number of γH2A.X foci per cell. These results correspond to previous studies using iron [63,64]. When a double-strand DNA break is induced by oxidative stress, there is a cascade of signalling (DNA damage response, DDR) which can result either in repair of the damage or senescence and/or apoptosis depending on the severity of damage. During DDR, a number of proteins are mobilized and co-located at the site of the damage. The detection by immunofluorescence of these different proteins makes it possible to indirectly visualize the double-strand breaks in the form of nuclear foci. For example, the ATM protein gets activated by DNA damage and then phosphorylates a multitude of proteins, including serine 139 of histone H2A.X allowing the attachment of MDC1 (mediator of DNA damage checkpoint protein 1) [65], which then guides the recruitment of repair proteins [66]. It has been demonstrated that iron-induced DNA damage [67,68,69] activates the DDR, including the ATM signalling pathway [70]. However, there was a difference between CSO and CO in the capability to decrease DNA damage in hepatic tissue under iron-overload conditions. The CSO showed a significant decrease in focus numbers compared to control values suggesting an antioxidant effect. However, we do not know whether CSO prevented the occurrence of DNA damage or promoted DNA repair. This topic has to be investigated in more detail in the future. In contrast, CO did not show any protective effect on the level of DNA damage. Others have demonstrated that the acetone extract from *Opuntia ficus-indica* cladodes decreased lipid peroxidation and DNA fragmentation in sperm cells during liquid storage [71]. The suggested antioxidant potential of CSO could be due to the high content of spinasterol and schottenol in the unsaponifiable fraction [18], which are absent in CO [15]. The reduction in the number of DNA damage foci by CSO could also be caused by the ability of schottenol and spinasterol to prevent the generation of ROS and the downstream DNA damage resulting in a decreased number of nuclear γH2A.X foci.

Given the importance of measuring total iron content in all three organs, we see this as an important perspective for future research. However, the findings we present still offer a significant contribution to the understanding of iron distribution patterns. This study lays a strong foundation for future work, which can build on our results by incorporating more detailed iron quantification across tissues, thereby deepening our collective knowledge of iron homeostasis.

## 4. Materials and Methods

### 4.1. Chemicals and Reagents

All chemicals were purchased from Sigma Aldrich (Saint Louis, MO, USA) unless otherwise stated.

### 4.2. Origin and Extraction of Oils

Cactus seed oil (from *Opuntia ficus-indica*) was provided by the cooperative of Sabbar Rhamna (Skhour Rhamna, Morocco), and colza oil (from Brassica napus subsp. Napus) from a commercial supermarket (Casablanca, Morocco). Cactus seed oil extraction was performed mechanically. An industrial prickly pear juice extracting machine (Philips Viva HR1832/00, Mumbai, India) was used to separate seeds and juice. After measuring its pH, the juice was preserved at −20 °C for further use, while seeds were washed, air dried and extracted by a cold press machine (Longer machinery, LGYL-80A, Zhengzhou, China). Both oils were stored in darkness until analysis and used within one month after purchase.

### 4.3. Identification of Opuntia ficus-indica Seed Oil

The 2 samples were solubilized in chloroform as solvent. The profile of volatile compounds was characterized by gas chromatography (GC) (Agilent, Santa Clara, CA, USA, 7890A Series) coupled with mass spectrometry (MS) equipped with a multimode injector and a column of 123-BD11 with dimensions of 15 m × 320 μm × 0.1 μm. The 4 μL of the soluble extract was injected into the column by 1/4 split mode using helium as carrier gas at a flow rate of 3 mL min^−1^ vector gas. The temperatures of the ion source and quadrupole were 230 °C and 150 °C, respectively. The oven temperature program was started at 30 °C and finished at 360 °C. The compounds identification was performed using NIST 2014 MS Library (Gaithersburg, MD, USA).

### 4.4. Animals and Experimental Design

Swiss OF1 male mice between 12 and 16 weeks of age were purchased from IFFA CREDO in Casablanca, Morocco. Mice were used under the recommendations of the Organization for Economic Co-Operation and Development (OECD), (Test no. 407: Repeated Dose 28-Day Oral Toxicity Study in Rodents, 1995). Experiments were carried out following the instructions of the Institutional Animal Ethics Committee of Hassan First University of Settat, Morocco, according to the National Institutes of Health Guide for the Care and Use of Laboratory Animals (NIH publication No. 85-23, revised 1985). All efforts were made to minimize animal suffering, and the number of animals used. Mice were acclimatized to our laboratory conditions for ten days in a pathogen-free environment at 22 ± 2 °C and a constant light-dark cycle (12 h-12 h). After acclimatization, mice were randomly divided into six groups (n = 3–4 mice/group) and fed with standard lab chow and water ad libitum. All efforts were made to minimize animal suffering, and the number of animals used. Each oil was solubilized in acetone as a solvent (1:4 *v*/*v*) and added to diet pellets. The concentration of iron used in our study was 3.5 µg Fe^2+^/mL. This concentration exceeded the average concentration of this chemical found in drinking water according to the World Health Organization (WHO), which is less than 0.3 mg/L [72], by 10 times. In addition, the administration of iron to mice was sustained over the entire 28-day treatment period. Before the treatment (Table 2), the diet was kept under a hood to evaporate the acetone, which served as the diluent for the oils. Six groups of mice were distributed and treated for 28 days as follows: Group I: a standard diet (Control) supplemented with acetone. Group II: a standard diet supplemented with 6% of Cactus oil (CSO) solubilized in acetone. Group III: a standard diet supplemented with 6% of Colza oil (CO) solubilized in acetone. Group IV: The reference drug Tardyferon (iron sulfate 3.5 µg Fe^2+^*/*mL) was dissolved in drinking water. Group V: Mice received iron and CSO. Group VI: Mice received iron and CO. For 28 days, the mice were weighed weekly using an electronic balance and no changes in body weights were found. After euthanasia, the liver, kidney and brain tissues were frozen in a dry ice bath and stored at −80 °C for further analysis.

### 4.5. Preparation of Homogenates

The 10% (*w*/*v*) liver, kidney and brain homogenates were prepared by grinding tissues. Homogenates were prepared by grinding the animals’ livers, brains and kidneys in 10% (*w*/*v*) of 50 mM potassium phosphate buffer (KH_2_PO_4_; K_2_HPO_4_; pH 7.4). Tissues were homogenized using a Potter–Elvehjem homogenizer. Homogenates were centrifuged for 10 min at 3000× *g* at 4 °C and supernatants considered as the crude cell-free extract and small aliquots were stored at −20 °C until further analyses.

### 4.6. Protein Content

Protein content was measured using bovine serum albumin as a standard, according to the method described by [73]. The absorbance was read spectrophotometrically at 750 nm (infinite M200 PRO, TECAN, Zürich, Switzerland).

### 4.7. Lipid Peroxidation

The level of MDA was measured as described by [74]. Under acidic conditions and at high temperature, two molecules of the thiobarbituric acid (TBA) react with one molecule of MDA forming a pink-coloured MDA-TBA2 adduct. This is a strongly visible light-absorbing molecule (532 nm). In addition, n-butanol is used to remove any remaining haemoglobin that could cause a false positive result due to haemoglobin’s absorbance at 540 nm, which is close to the adduct’s absorbance. The reaction mixture contained 500 µL of homogenate, 500 µL of trichloroacetic acid (20%) and 1 mL of TBA (0.67%). The mixture was heated at 100 °C for 15 min. After cooling in an ice bath, 4 mL of n-butanol was added. After centrifuging at 3000× *g* for 15 min, the absorbance of the organic layer at 532 nm was measured. MDA level was expressed as nanomoles of MDA per milligram of protein.

### 4.8. Gluthatione

The content of GSH was measured as described by the method described by [75]. The sulfhydryl reagent 5,5′-dithio-bis (2-nitrobenzoic acid) (DTNB) and GSH react to generate the yellow-coloured derivative 5′-thio-2-nitrobenzoic acid (TNB). The reaction mixture contained 200 µL trichloroacetic acid (TCA) (5%) and 400 µL homogenate. After centrifuging at 12,000× *g* for 10 min, 50 µL of supernatant was added to 100 µL of DTNB (6 mM) and 850 µL of 50 mM phosphate buffer. After 5 min, the absorbance was read spectrophotometrically at 412 nm. The GSH level was expressed as nanomoles of GSH per milligram of protein.

### 4.9. Superoxide Dismutase

The activity of SOD was assessed by the method of [76]. This method involves the illumination of riboflavin in the presence of EDTA, which triggers a reduction in flavin. Subsequently, it oxidizes and reduces oxygen to a superoxide anion. This radical reacts with Nitro Blue Tetrazolium (NBT), reducing the NBT to a blue-coloured formazan product. The presence of total SOD activity in the sample inhibits the production of formazan, resulting in a decrease in the intensity of the formed formazan, which is measured at 560 nm in a spectrophotometer. The reaction mixture contained 20 µL of tissue homogenate, 50 mM phosphate buffer, 0.025% triton x-100, 0.1 mM EDTA pH 8, 12 mM L-methionine, 75 mM NBT, homogenate, and 2 µM riboflavin. Then, the mixture was thoroughly mixed and placed 30 cm below a light source comprising a 15 W fluorescent lamp for 10 min.

### 4.10. Gluthatione Peroxidase

The activity of total GPx was assessed by the method of [77]. The method involves the ability of the GPx enzyme to oxidize GSH by the sulfhydryl reagent 5,5′-dithiol-bis (2-nitrobenzoic acid) (DTNB) to form a yellow-coloured derivative 5′-thio-2-nitrobenzoic acid (TNB). The formed TNB is measured at 412 nm in a spectrophotometer. The reaction mixture contained 200 µL of the tissue homogenate, 400 µL potassium phosphate buffer (0.4 mM, pH 7.0), 200 µL GSH (2 mM), 200 µL of EDTA (0.8 mM), 100 µL sodium azide (10 mM) and 100 µL of H_2_O_2_ (2.5 mM). The mixture was incubated for 10 min at 37 °C. Afterwards, the reaction was stopped by TCA (10%) and centrifuged at 2000× *g* for 5 min. Then, 2.5 mL of sodium phosphate tribasic (0.1 M) and 1 mL of DTNB buffer (0.04%) were added to the supernatant and absorbance determined spectrophotometrically.

### 4.11. Immunofluorescence Analysis

#### 4.11.1. Dewaxing and Antigen Retrieval

Sections (5 µm) of paraffin-embedded hepatic tissue were incubated at 60 °C for 30 min, dewaxed in Histoclear for 2 × 10 min, then rehydrated in 100% I, 100% II, and 90% and 70% ethanol for 10 min each. The slides were immersed in distilled water for 2 × 5 min. Then, they were placed in the microwave in citrate buffer at high power and 40% power for 4 min and 10 min, respectively.

#### 4.11.2. Immunofluorescence Staining

Immunofluorescence staining on paraffin-embedded sections of liver was performed as described previously by [78]. After being washed in PBS, samples were blocked with 5% normal goat serum (NGS) and 1% BSA in PBS for 60 min at room temperature. Samples were rinsed and blocked with avidin for 15 min. Samples were then washed and blocked with biotin for 15 min. After being rinsed, samples were incubated overnight with rabbit monoclonal primary antibody to γH2A.X (#9718, Cell Signaling Technologies, Danvers, MA, USA) diluted in blocking solution at 4 °C. Samples were washed three times in PBS for 5 min each. Samples were incubated in biotinylated secondary antibody diluted in blocking solution for 30 min. Samples were washed three times in PBS for 5 min each. Samples were then incubated with Avidin diluted in PBS for 30 min. The samples were then washed three times in PBS for 5 min each. Nuclei were stained with DAPI and sections washed in PBS for 10 min. Samples were mounted in Vectashield anti-fading solution (Vector Laboratories, Newark, NY, USA). Images were visualized and captured under a fluorescence microscope (Leica DMi8, Leica Microsystems, Wetzlar, Germany), and then analysed using Image J (https://imagej.nih.gov/ij/, accessed on 17 September 2024). Five images were taken under the microscope at different fields of view for each sample/mouse. The focus counting was performed manually using image J.

### 4.12. Statistical Analysis

GraphPad Prism 8 (GraphPad Software, Inc., San Diego, CA, USA) was used for statistical analysis. All results were expressed as the average of mean ± standard deviation, except for DNA Damage, where the results were expressed as the average of mean ± standard error of the mean. Statistics were executed using a two-way ANOVA followed by a Tukey’s test for multiple comparisons. A difference in the mean values of *p* ≤ 0.05 was considered to be statistically significant.

## 5. Conclusions

Our study suggests that cactus seed oil from *Opuntia ficus-indica* has the capacity to alleviate selected parameters of iron-overload-induced oxidative stress such as MDA and GSH in the liver and brain, respectively, while no effects were found in the kidney. In contrast, it unexpectedly decreased antioxidant capacities of SOD and GPX in the kidney and brain, respectively, which requires further investigation to better understand the underlying molecular mechanisms. Importantly, CSO treatment was able to significantly reduce the amount of DNA damage in the liver, underlining its protective effect. Together, our results provide encouraging data on some beneficial effects of CSO against several oxidative stress parameters in selected tissue types. In the future, combining lipidomic and transcriptomic analysis would elucidate the metabolic signalling pathways involved in the CSO reno-, neuro- and hepatoprotective effects against iron overload. This approach may bring new arguments to the potential benefits of CSO in managing of renal, neuronal and hepatic pathologies associated with iron overload and oxidative stress. Additionally, this may document the potential beneficial role of CSO in lowering the deleterious effects of oxidative stress and as a new therapeutic option with fewer adverse effects than synthetic compounds.

## Figures and Tables

**Table 1:** Composition of oils.

	Cactus Oil	Colza Oil
**Fatty Acids (%)**		
Myristic acid C14:0	0.14	0.12
Palmitic acid C16:0	13.85	6.48
Oleic acid C18:1	9.95	77.18
Linoleic acid C18:2	67.32	
Stearic acid C18:0	4.61	1.57
Arachidic acid C20:0	0.58	2.38
Eicosenoic acid C20:1	1.10	4.84
Behenic acid C22:0	0.28	1.32
Erucic acid C22:1	0.42	0.37
Lignoceric acid C24:0	0.26	0.46
Nervonic acid C24:1	0.07	0.49
Pentacosylic acid C25:0	0.03	
Cerotic acid C26:0	0.05	
**Phytosterol (%)**		
Campesterol	0.10	1.35
γ-sitosterol	0.92	1.77
α.1-Sitosterol	0.03	
1,8,11-Heptadecatriene	0.13	
Stigmasta-5,24(28)-dien-3-ol	0.07	
**Tocopherol (%)**		
γ-tocopherol	0.08	0.15

**Table 2:** Conditions of treatment.

Groups	Treatment
Group I	A standard chow with the soluent acetone
Group II	A standard chow (as Group I) supplemented with 6% of CSO solubilized in acetone
Group III	A standard chow (as Group I) supplemented with 6% of CO solubilized in acetone
Group IV	The damaging agent Tardyferon (iron sulphate 3.5 µg Fe^2+^/mL) was dissolved in drinking water
Group V	The animals received iron (as Group IV) and CSO (as Group II)
Group VI	The animals received iron (as Group IV) and CO (as Group III)

## Data Availability

Data is contained within the article.
